# Differential regulation of *Shigella* Spa47 ATPase activity by a native C-terminal product of Spa33

**DOI:** 10.3389/fcimb.2023.1183211

**Published:** 2023-06-14

**Authors:** Heather B. Case, Saul Gonzalez, Marie E. Gustafson, Nicholas E. Dickenson

**Affiliations:** Department of Chemistry and Biochemistry, Utah State University, Logan, UT, United States

**Keywords:** bacterial pathogenesis, ATPase, injectisome, type III secretion apparatus (T3SA), type III secretion system (T3SS), *Shigella*, Spa47, virulence factor

## Abstract

*Shigella* is a Gram-negative bacterial pathogen that relies on a single type three secretion system (T3SS) as its primary virulence factor. The T3SS includes a highly conserved needle-like apparatus that directly injects bacterial effector proteins into host cells, subverting host cell function, initiating infection, and circumventing resulting host immune responses. Recent findings have located the T3SS ATPase Spa47 to the base of the *Shigella* T3SS apparatus and have correlated its catalytic function to apparatus formation, protein effector secretion, and overall pathogen virulence. This critical correlation makes Spa47 ATPase activity regulation a likely point of native control over *Shigella* virulence and a high interest target for non-antibiotic- based therapeutics. Here, we provide a detailed characterization of the natural 11.6 kDa C-terminal translation product of the *Shigella* T3SS protein Spa33 (Spa33C), showing that it is required for proper virulence and that it pulls down with several known T3SS proteins, consistent with a structural role within the sorting platform of the T3SS apparatus. *In vitro* binding assays and detailed kinetic analyses suggest an additional role, however, as Spa33C differentially regulates Spa47 ATPase activity based on Spa47s oligomeric state, downregulating Spa47 monomer activity and upregulating activity of both homo-oligomeric Spa47 and the hetero-oligomeric MxiN_2_Spa47 complex. These findings identify Spa33C as only the second known differential T3SS ATPase regulator to date, with the *Shigella* protein MxiN representing the other. Describing this differential regulatory protein pair begins to close an important gap in understanding of how *Shigella* may modulate virulence through Spa47 activity and T3SS function.

## Introduction

Diarrheal disease is a significant worldwide health concern, responsible for 1.6 million deaths in 2017 alone and an alarming one in nine deaths in children under the age of 5 (Bernadeta Dadonaite HRaMR, 2020). Diarrhea is a symptom of infections by a range of parasites, viruses, and bacteria, with rotavirus, *E. coli*, *cryptosporidium*, and *Shigella* responsible for the majority of reported cases (Keusch et al., 2006). Infection by the Gram-negative bacterial pathogen *Shigella* causes an exceptionally severe form of diarrheal disease (bacillary dysentery) that alone is responsible for an estimated 90 million infections and 100,000 deaths annually (Kotloff et al., 2013). Like many diarrheal pathogens, *Shigella* is transmitted through the fecal oral route and is therefore most common in developing regions where access to clean drinking water, waste stream treatment, and medical care are limited. However, *Shigella* infections are consistently reported throughout the developed world as well (Taneja and Mewara, 2016). Furthermore, the recent emergence of multidrug-resistant *Shigella* strains and its incredibly low infectious dose (10–100 bacteria) (DuPont et al., 1989; Niyogi, 2005) has “earned it a place” on the World Health Organization’s (WHO) priority pathogens list for those in highest need of research and development of new therapeutics (World Health Organization, 2017). Other bacterial pathogens named on this list include *E. coli*, *Pseudomonas*, and *Salmonella*, which, like *Shigella*, all rely on one or more type three secretion systems (T3SS) to initiate and sustain infections.

Structurally, all T3SSs include a needle and syringe-like apparatus (injectisome) that is anchored to the bacteria by a basal body that spans both the inner and outer membranes, providing support for the apparatus and a dedicated path across both membranes (Erhardt et al., 2010). A narrow cylindrical needle structure protrudes from the basal body with a tightly regulated length that extends beyond the lipopolysaccharide layer (Mota et al., 2005; West et al., 2005). The needle terminates in a multi-protein tip complex that senses unique environmental cues relevant to the infection pathway of the bacterium and forms the translocon pore that penetrates a contacted host cell membrane, completing the unidirectional conduit between the bacteria and host cell cytoplasm (Epler et al., 2009; Dickenson et al., 2011). Recently, high-resolution fluorescence and Cryo-ET imaging have begun to uncover structural details of the cytoplasmic sorting platform that resides at the base of the T3SS injectisome (Hu et al., 2015; Lunelli et al., 2020; Tachiyama et al., 2021). Structural and biochemical assessment of the sorting platform suggests that it is responsible for recognizing and partially unfolding secreted protein substrates and that ATP hydrolysis by a homo-hexameric T3SS ATPase located within the sorting platform is required for these events to proceed efficiently (Akeda and Galan, 2005; Burgess et al., 2016a; Majewski et al., 2019). The specific mechanism(s) through which the ATPase supports T3SS function remains somewhat unclear, although recent work in several T3SSs suggest that it works in conjunction with an electrochemical gradient across the inner membrane to overcome the thermodynamic barrier associated with chaperone release, substrate unfolding, and transit though the narrow T3SS needle (Wagner et al., 2018; Minamino et al., 2022). Loss of function and knockout mutations to the *Shigella* ATPase result in incomplete apparatus formation, lack of effector protein secretion, and avirulent mutant bacterial strains (Burgess et al., 2016a; Burgess et al., 2016b; Burgess et al., 2020), making Spa47 a highly sought after target for small molecule therapeutic treatments and a likely point of regulation for *in vivo* control of protein secretion and virulence.

In fact, *in vivo* regulation of T3SS activity *via* interaction of the T3SS ATPase with other T3SS proteins is supported by several examples in which these interactions modify ATPase activity *in vitro*. Specifically, the T3SS proteins FliH, YscL, and CdsL from *Salmonella*, *Yersinia*, and *Chlamydia*, respectively, each inhibit activity of their corresponding T3SS ATPases (Minamino and MacNab, 2000; Blaylock et al., 2006; Stone et al., 2011). The *Shigella* homolog to these regulators (MxiN) was also demonstrated to influence the activity of the *Shigella* T3SS ATPase, Spa47; however, MxiN additionally provides a previously unprecedented differential regulation of Spa47 ATPase activity based on Spa47’s oligomer state, downregulating oligomeric Spa47 activity while upregulating activity of monomeric Spa47 isolates (Case and Dickenson, 2018). This differential regulation was the only example of such for any T3SS ATPase and led us to hypothesize that MxiN plays a regulatory role in controlling ATPase activity of both cytoplasmic and injectisome-incorporated populations of Spa47.

We then hypothesized that interaction with additional T3SS proteins is likely required to provide opposite impact on Spa47 activity (compared to MxiN) and provide complete regulation capabilities for both cytoplasmic and sorting platform-incorporated Spa47 populations. One of the T3SS proteins of interest as a likely candidate for additional regulation roles is Spa33, as it has previously been shown to pull down with both Spa47 and MxiN (Morita-Ishihara et al., 2006). Until now, these interactions have been solely attributed to its structural role as a cytoplasmic ring (C-ring) component of the T3SS where interaction with the protein MxiK anchors the apparatus to the membrane components of the T3SS (Schuch and Maurelli, 2001; Morita-Ishihara et al., 2006; Tachiyama et al., 2019) and its interactions with MxiN appropriately locate the sorting platform components directly below the basal body and entrance to the needle (Tachiyama et al., 2021). Consistent with T3SS homologs from *P. syringae*, *Y. pestis*, and *S. enterica* (Fadouloglou et al., 2004; Bzymek et al., 2012; Lara-Tejero et al., 2019), Spa33 is natively expressed as both a full-length (Spa33FL) and C-terminal product resulting from an internal ribosome binding site (RBS) and non-canonical start codon (Spa33C) (McDowell et al., 2016; Kadari et al., 2019). There has been a great deal of interest in understanding the role of this and other recently identified native Spa33 truncations (Kadari et al., 2019), and as a result, it is understood that both Spa33FL and Spa33C are necessary for proper secretion of effector proteins through the *Shigella* T3SA (McDowell et al., 2016; Kadari et al., 2019), and it has more recently been suggested that hetero-oligomers of the full-length and abbreviated protein constructs may in fact fulfill the structural role in the sorting platform that was previously attributed solely to Spa33FL (Tachiyama et al., 2021).

In this study, we explore the characteristics and role(s) of the *Shigella* Spa33C construct with respect to biophysical properties, apparatus association, and potential T3SS regulatory mechanisms. We show that expression of both Spa33FL and Spa33C is necessary for proper *Shigella* effector/translocator protein secretion and host cell invasion phenotype and that both Spa33FL and Spa33C co-purify with the T3SS sorting platform proteins Spa47 and MxiN. Live cell total internal reflection fluorescence microscopy (TIRFM) imaging of fluorescent protein chimeras developed for this study further support that both constructs are capable of independently associating with the T3SS sorting platform. *In vitro* kinetic characterization uncovered a regulatory role for the Spa33C construct, with homo-dimeric Spa33C interacting strongly with both monomeric and oligomeric Spa47 populations and serving as a differential regulator of Spa47 ATPase activity based on Spa47 oligomer state. These findings help to uncover new and important T3SS regulatory mechanisms in *Shigella* and lays an important foundation for exploring T3SSs from other pathogens in search of similar regulatory opportunities.

## Materials and methods

### Materials

Wild-type *Shigella flexneri* corresponds to the serotype 2a 2457T strain originally isolated in 1954 (Formal et al., 1958). *Escherichia coli* strains and 2× ligation mix were from Novagen (Madison, WI). Restriction enzymes, the commercial pTYB21, pACYC, pRSF, and pET15b protein expression plasmids, polymerase chain reaction (PCR) buffer, Q5 High-Fidelity polymerase, and chitin resin were purchased from New England Biolabs (Ipswich, MA). Oligonucleotide primers were from Integrated DNA Technologies (Coralville, IA). HeLa cells were from the American Type Culture Collection (Manassas, VA). The Superdex 200 Increase 10/300 and 16/600 size exclusion columns, 1 and 5 ml HisTrap Crude FF, and 5 ml HiTrapQ FF columns were purchased from GE Healthcare (Pittsburgh, PA). ATP was from Sigma-Aldrich (St. Louis, MO). Dithiothreitol (DTT), isopropyl ß-D-1-thiogalactopyranoside, ampicillin, kanamycin, and chloramphenicol were from Gold Biotechnology (St. Louis, MO). The malachite green assay kit was purchased from BioAssay Systems (Hayward, CA). All other solutions and chemicals were of reagent grade.

### Invasions

Invasion phenotype of *S. flexneri* strains lacking the gene for Spa33, expressing wild-type Spa33, expressing only Spa33FL, expressing only Spa33C, and expressing both Spa33FL and Spa33C from a polycistronic vector were determined by a gentamicin protection assay. The protocol used here was first described by Niesel and coworkers (Niesel et al., 1985) and provides a powerful virulence phenotype assay sensitive to the presence of a functional T3SS. Briefly, sterile 24-well plates were seeded with passaged HeLa cells and grown overnight in Dulbecco’s modified Eagle’s medium (DMEM) supplemented with 10% fetal calf serum, penicillin, and streptomycin at 100% relative humidity, 37°C, and 5% CO_2_. The *S. flexneri* strains were streaked onto tryptic soy agar plates containing 0.025% Congo red and grown overnight at 37°C. Small cultures containing appropriate antibiotics were inoculated from the agar plates and grown to OD_600_ of ∼0.5 at 37°C and 200 rpm. Equivalent bacterial loads [multiplicity of infection (MOI) = 10] were introduced to the cultured HeLa cells and the plates were centrifuged at 1,000 × *g* for 5 min to synchronize contact between the bacteria and HeLa cells. The inoculated cells were incubated at 37°C for 30 min to allow invasion, rinsed to remove extracellular bacteria, and treated with 50 μg/ml gentamicin to selectively kill any remaining *Shigella* that had not successfully invaded the HeLa cells. The HeLa cells were then lysed with 1% agarose in water and overlaid with a Luria agar (Miller) solution. Overnight incubation at 37°C resulted in *Shigella* colony formation from the previously internalized bacteria, allowing a quantitative comparison of invasion phenotype among the tested *Shigella* strains.

### Protein expression and purification

The gene encoding Spa47 was purchased as a double-stranded gBlock product from Integrated DNA Technologies and cloned into the expression plasmid pTYB21, as described previously (Burgess et al., 2016b). The gene for MxiN was amplified from the *S. flexneri* 2457T strain and independently cloned into both pACYC and pET15b for expression in *E. coli*, as described previously (Case and Dickenson, 2018). The gene encoding Spa33 was amplified from the *S. flexneri* 2457T strain and cloned into the second cloning site of pRSF for expression in *E. coli* and into pWPsf4 for expression in *Shigella*. The gene for Spa33FL was generated by making two point mutations, one in the internal RBS and one in the internal/alternative start codon, to ensure that only the full-length Spa33 product is expressed, as first described by Mcdowell et al (McDowell et al., 2016). The DNA sequence that encodes for Spa33C was amplified from the wild-type sequence to generate the Spa33C construct, with the internal/alternative GTG start codon mutated to the canonical ATG for optimal expression levels. N-terminal enhanced green fluorescent protein (EGFP) chimeras of both Spa33FL and Spa33C were engineered by including the DNA sequence encoding EGFP and a short linker sequence (GSAASENLYFQS) 5’ to the multiple cloning site of pWPsf4 (synthetic construct purchased from General Bioscience, Brisbane, CA). The *spa33C* and *spa33FL* genes were inserted 3’ to *EGFP* (in the pWPsf4 MCS) for protein expression in *Shigella*. The *spa33* and *EGFP-spa33* genes were additionally cloned into the two cloning sites of the engineered polycistronic pWPsf4 vector to support transcription and translation of the required combinations of Spa33 constructs. *EGFP-spa33C* and *EGFP* alone were each cloned into pET15b to allow expression and purification from *E. coli* via an N-terminal 6 x histidine tag. All constructs were sequence verified by Sanger sequencing (Genewiz, Inc., South Plainfield, NJ).

All Spa47, MxiN, Spa33, and EGFP constructs in *E. coli*, including co-expressions, were expressed as follows: *E. coli* cells containing the appropriate plasmid(s) were streaked onto an LB agar plate containing the required antibiotic(s) and incubated overnight at 37°C. This plate was used to start liquid Terrific Broth (TB) cultures containing appropriate antibiotics and grown to an OD_600_ of 0.8 at 37°C and 200 rpm. The cultures were then cooled to 17°C prior to induction with 1 mM isopropyl β-d-1-thiogalactopyranoside (IPTG). Following induction, cultures remained at 17°C and 200 rpm for 20 h prior to harvesting the cultures.

Recombinantly expressed Spa33C, EGFP-Spa33C, and EGFP were each purified *via* immobilized metal affinity chromatography (IMAC) *via* N-terminal 6 x histidine tags. Cells were harvested by centrifugation followed by resuspension in binding buffer [20 mM Tris, 500 mM NaCl, 5 mM imidazole, and 5% (v/v) glycerol (pH 7.9)] containing 0.2 mM protease inhibitor 4-(2-aminoethyl) benzenesulfonyl fluoride hydrochloride (AEBSF), and membrane disruption was accomplished by sonication. The sonicated suspension was clarified by centrifugation, and the soluble protein was purified using a GE HisTrap Crude FF IMAC affinity column connected to an ÄKTA FPLC system and eluted using an imidazole gradient. IMAC elution fractions containing purified Spa33C or EGFP were assessed by sodium dodecyl sulfate–polyacrylamide gel electrophoresis (SDS-PAGE), pooled, and further purified using a Superdex 10/300 sizing column equilibrated with 20 mM Tris, 100 mM NaCl, 5 mM DTT, and 5% (v/v) glycerol (pH 7.9). Final concentrations for Spa33C were determined using in gel densitometry of Coomassie-stained acrylamide gels and bovine serum albumin (BSA) as a standard. Concentrations of EGFP and EGFP constructs were calculated using the published molar extinction coefficient of EGFP (56,000 M^−1^ cm^−1^ at 484 nm) (Soleja et al., 2018).

Spa47 was purified as described previously (Burgess et al., 2016b). Following isolation from the culture media, the *E. coli* cells were resuspended in 20 mM Tris and 500 mM NaCl (pH 7.9) containing 0.2 mM 4-(2-aminoethyl)benzenesulfonyl fluoride hydrochloride (AEBSF), lysed by sonication, and purified using a chitin affinity column. On-column cleavage of the intein linker domain by exposure to 50 mM DTT eluted Spa47 protein from the chitin column at relatively high purity. The eluted Spa47 was then further purified by negative selection over an anion exchange column and finally purified/characterized using a Superdex 200 16/600 size exclusion column equilibrated with 20 mM Tris, 100 mM NaCl, and 5 mM DTT, pH 7.9. Spa47 concentrations were determined using in-gel densitometry of Coomassie-stained acrylamide gels and BSA as a standard, as previously described (Burgess et al., 2016b). All Spa47 concentrations are reported in monomer concentration units for consistency and clarity.

### Characterization of Spa33C/Spa47 interactions

EGFP-Spa33C supported fluorescence polarization measurements to observe binding between Spa33C and both monomeric and oligomeric Spa47 isolates. These binding events were observed and the affinities measured by holding the EGFP-Spa33C concentration constant at 100 nM, while the concentration of Spa47 was titrated from 0–1.5 μM. The polarization values were collected using a Synergy H4 fluorescence plate reader equipped with motorized polarizers and appropriate filter sets. The change in polarization relative to that of EGFP-Spa33C alone was plotted as a function of Spa47 (monomer or oligomer) concentration. The substrate concentration resulting in the half maximum change in polarization was determined by fitting the data to a single site saturation binding model in SigmaPlot 12. A control condition testing for binding between purified EGFP and Spa47 was additionally performed to ensure that the observed binding between Spa33C and Spa47 results from specific interactions between the intended T3SS proteins and is not driven by interactions involving EGFP. Importantly, we have previously shown that isolated monomeric and oligomeric populations of Spa47 are in equilibrium; however, each species is stable for several days following SEC isolation (Burgess et al., 2020). Expeditious use of the isolates ensured that not only polarization but also all described assays are performed on the intended Spa47 oligomer states.

### ATP hydrolysis activity assay

Spa47 ATPase activity was measured using a malachite green assay kit according to manufacturer guidelines. The effect of Spa33C on the rate of ATP hydrolysis was examined on SEC-isolated Spa47 oligomer, monomer, and co-expressed MxiN_2_Spa47 complex that was identified in a prior study (Case and Dickenson, 2018). The concentration of the Spa47 complex was held constant at 0.05, 0.15, and 0.15 μM for oligomeric Spa47, monomeric Spa47, and MxiN_2_Spa47, respectively, and exposed to increasing concentrations of Spa33C (0, 1, 2.5, 5, and 10 μM). Additionally, Spa47 oligomer, monomer, and MxiN_2_Spa47 complex activities were tested in the presence of BSA in place of Spa33C (1, 5, and 10 μM) as a control to ensure that the observed influences on activity were not the result of molecular crowding or non-specific protein interactions. Substrate-dependent kinetic analyses were additionally performed on each Spa47 species at the same concentration previously described (both in the presence and absence of 4 μM Spa33C). Initial reaction velocities were determined for 0.025, 0.075, 0.150, 0.30, and 0.60 mM substrate (ATP) and plotted as the initial velocity versus ATP concentration. SigmaPlot 12 was used to fit each data set to the Michaelis–Menten equation:


(**Equation 1**) $$v=\frac{V_{max}\left[S\right]}{K_{M}+\left[S\right]}$$

where v is the initial velocity of the reaction, [S] is the ATP concentration, K_M_ is the Michaelis constant, and Vmax is the maximum velocity of the enzyme.

### Live-cell TIRFM detection and localization of fluorescent Spa33 constructs

*Shigella flexneri* expressing EGFP-Spa33 were grown to an OD_600_ of ~1.0. Ten milliliters of each culture were centrifuged at 2,272 × g for 4 min to gently pellet the bacteria. The bacteria were washed once with room temperature phosphate-buffered saline (PBS) and then resuspended in 500 μl of PBS and diluted as necessary to achieve the desired density of bacteria in the microscopy images. Twenty microliters of the diluted bacteria were placed on a No. 1.5 cover slide, and fluorescence images were collected using an Olympus IX51 total internal reflection fluorescence microscope (TIRFM) (Olympus, Center Valley, PA) equipped with a 100× TIRF objective (1.49 NA, infinity corrected, achromat). The 488 nm laser line of a Toptica Chrome laser system (Toptica Photonics, Farmington, NY) was directed through the objective and the sample fluorescence collected in epifluorescence geometry and filtered through appropriate dichroic mirrors and bandpass filters prior to collection on a Hamamatsu ORCA-Flash4.0 CMOS camera (Hamamatsu, Hamamatsu City, Japan). Excitation parameters and image collection were controlled with cellSense Dimension software (Olympus, Center Valley, PA).

## Results

### Expression of both Spa33FL and Spa33C is required for *Shigella* virulence

**Figure 1:**
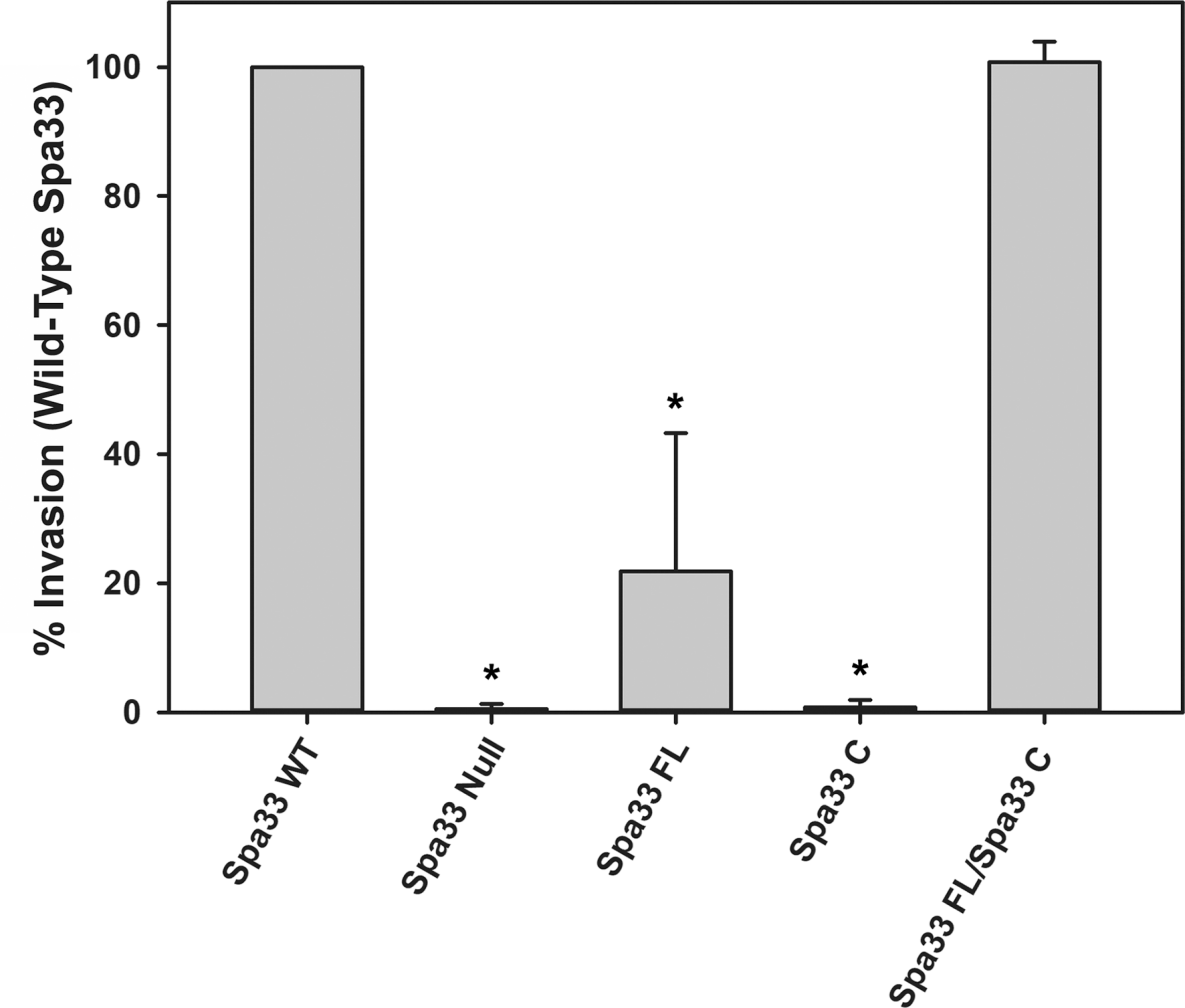
Effect of Spa33 variants on *Shigella* invasion phenotype. The ability of the *Shigella* mutants to invade eukaryotic host cells was measured by a standard gentamicin protection assay. Invasion results are presented as the percentage of invasion by an *S. flexneri* strain expressing wild‐type Spa33 and represent at least three independent experimental data sets. Data are presented as mean values ± the standard deviation. *Indicates a significant difference in invasion levels between the wild-type Spa33 strain and the specified mutant (one-way ANOVA followed by a Dunnett’s post-test, p ≤ 0.05)).

It was recently shown that Spa33 naturally expresses as at least two distinct variants in the cell, namely, the 293-amino- acid full-length protein (Spa33FL) and a 102- amino- acid C-terminal product that results from an internal ribosome binding site and an “in- frame” alternative start codon (Spa33C) (McDowell et al., 2016; Kadari et al., 2019). Both constructs appear to be biologically important, as *Shigella* strains that do not express either the full length or the truncated Spa33 constructs display little/no T3SS activity, as they are unable to appropriately secrete effector proteins in a Congo red protein secretion assay (McDowell et al., 2016) or form pores in red blood cell membranes in a hemolysis assay (Tachiyama et al., 2021). To better understand the cooperative effect of these Spa33 constructs in the T3SS and *Shigella* virulence and to validate the Spa33FL and Spa33C constructs and polycistronic expression vector engineered for this study, a gentamycin protection (cellular invasion) assay was performed. Cellular invasion phenotype was evaluated for a Spa33 null *Shigella* strain unable to express either Spa33 construct and *Shigella* strains expressing: 1) both Spa33FL and the truncated Spa33C constructs from the wild-type *spa33* gene sequence containing an internal RBS and start codon, 2) a strain expressing only Spa33FL (the internal RBS and start codon were mutated to maintain appropriate amino acid sequence but prevent expression of the Spa33C product, 3) a strain expressing only Spa33C (only the sequence corresponding to the Spa33C product was cloned into the expression vector), and 4) a strain with a polycistronic pWPsf4 expression plasmid containing genes for constitutive simultaneous expression of both Spa33FL and Spa33C under the control of a single promotor/terminator pair and utilizing identical ribosome binding site sequences located upstream from each gene. As seen in **Figure 1**, the Spa33-null and Spa33C-only strains were essentially non-invasive, with the strain expressing only Spa33FL recovering 21.8 ± 21.4% wild-type invasion levels. The polycistronic strain expressing both Spa33FL and Spa33C restored invasion to wild-type levels (100.7 ± 3.2%). An effector secretion profile (IpaC) of these strains tracks well with the invasion results, confirming that the invasion phenotype reliance on expression of both constructs correlates to T3SS function (**Supplementary Figure S1**). Together, these findings validate both the engineered Spa33 constructs and the polycistronic expression vector and provide the first demonstration that recombinant expression of Spa33FL and Spa33C reinstates T3SS function/*Shigella* virulence in a Spa33 null strain, underscoring the importance of each construct in T3SS activity and *Shigella* virulence.

### Spa33FL and Spa33C co-purify with Spa47 and MxiN

**Figure 2:**
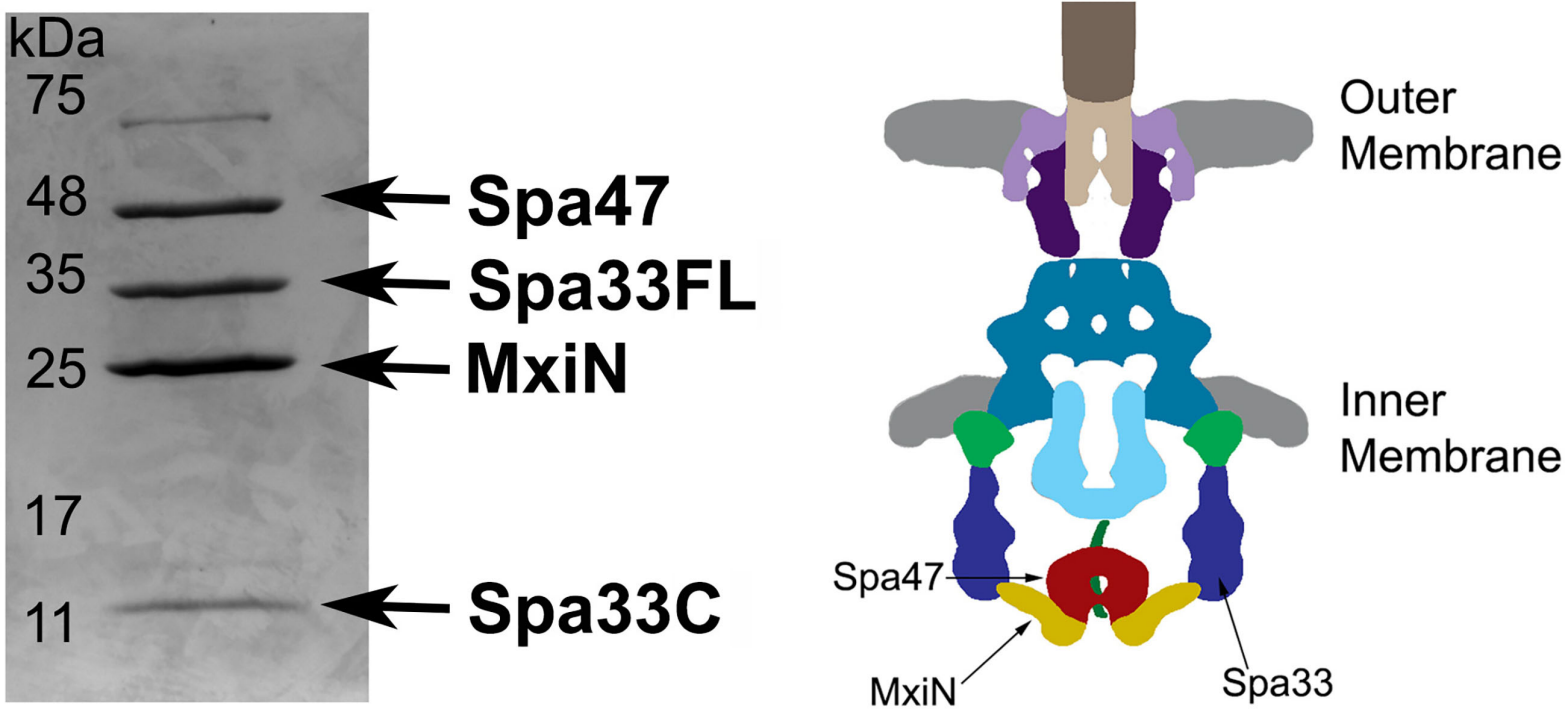
Spa33FL and Spa33C interact with Spa47 and MxiN. Co-expressed Spa47, MxiN, and Spa33WT were purified from *E. coli via* chitin- tagged Spa47 over a chitin column. Elution fractions showed four distinct bands matching in size and verified through mass spectrometry to be Spa47, Spa33FL, MxiN, and Spa33C. A diagram of the base of the type three secretion system is shown with the predicted locations of Spa47, MxiN, and Spa33 identified for reference.

Based on Cryo-ET maps of the *Shigella* sorting platform and previous mass spectrometry analysis of isolated wild-type Spa33 complexes (McDowell et al., 2016; Tachiyama et al., 2021), it has been suggested that two heterotrimeric Spa33FL/Spa33C_2_ complexes are present in each of the six sorting platform pods, locating MxiN and Spa47 at the base of the platform. To further explore this relationship, Spa33WT (WT gene sequence capable of expressing both Spa33FL and Spa33C) was co-expressed and co-purified with MixN and Spa47. The putative complex was purified *via* an N-terminal chitin binding domain and intein cleavage site on Spa47 as previously described (Burgess et al., 2016b). The complex eluted from the chitin column displayed four distinct bands on SDS-PAGE (**Figure 2**). Two of the bands correspond to Spa47 and MxiN, which have previously been identified through mass spectrometry (Case and Dickenson, 2018). The other two bands were approximately 30 and 11 kDa, and when analyzed via mass spectrometry, both were identified as Spa33, with the 30-kDa band corresponding to Spa33FL and the 11-kDa band corresponding to Spa33C. The faint band at approximately 70 kDa is *E. coli* Hsp70, a common contaminant observed during Spa47 chitin purification. In addition, a pull-down assay with purified Spa47 (containing no purification tag) and Spa33C (containing an N-terminal 6 x histidine tag) was performed. A modest amount of Spa47 robustly pulled down with Spa33C, demonstrating a direct interaction between Spa47 and Spa33C that is independent of additional sorting platform components (**Supplementary Figure S2**).

### Biophysical characterization of Spa33C

Now, understanding that the naturally expressed Spa33C construct is critical to proper T3SS function and *Shigella* virulence, that it co-purifies as a part of a complex containing the sorting platform proteins Spa33FL, MxiN, and the ATPase Spa47, and that it can interact directly with Spa47, we assessed several biophysical properties of Spa33C. It is worth noting that the exclusively full-length Spa33 construct was successfully expressed in *E. coli*; however, it remained insoluble despite significant efforts to extract it from inclusion bodies and solubilize the protein, preventing it from further consideration in this study. This is consistent with previous observations and with the suggestion that Spa33FL solubility in *Shigella* is supported by interactions with additional T3SS proteins, including Spa33C (McDowell et al., 2016; Kadari et al., 2019; Tachiyama et al., 2021). On the other hand, we were able to purify ample amounts of the soluble Spa33C construct to characterize it by far-UV circular dichroism (CD) and analytical ultracentrifugation (AUC). **Supplementary Figure S3** shows the far-UV CD spectra and thermal melt profile for the recombinantly expressed and purified Spa33C utilized in this study. The CD spectra are consistent with the published 2.30 Å crystal structure (McDowell et al., 2016) and recent spectroscopic data published during the preparation of this manuscript (Tachiyama et al., 2021), illustrating a strong negative peak at 208 nm that is indicative of significant β-sheet content. **Supplementary Table S1** includes the calculated percent secondary structure content values based on the collected spectra and the calculated melt temperature (Tm) obtained from the CD thermal unfolding profile. The sharp thermal transition observed at 77 ± 1°C supports a highly stable protein experiencing a catastrophic thermal unfolding event that affects the global secondary structure.

The purified Spa33C construct was additionally characterized by sedimentation velocity analytical ultracentrifugation (SV-AUC) to assess the stoichiometry and overall distribution of Spa33C complexes present in the solution (**Supplementary Figure S4**). The AUC analysis clearly identifies a single dominant species in the solution with a sedimentation coefficient that corresponds to a calculated molecular weight of 27 ± 3 kDa, in excellent agreement with that of a homodimer of Spa33C containing an N-terminal His tag (dimer = 27.5 kDa).

### Spa33C homodimers interact strongly with both monomeric and oligomeric Spa47

**Figure 3:**
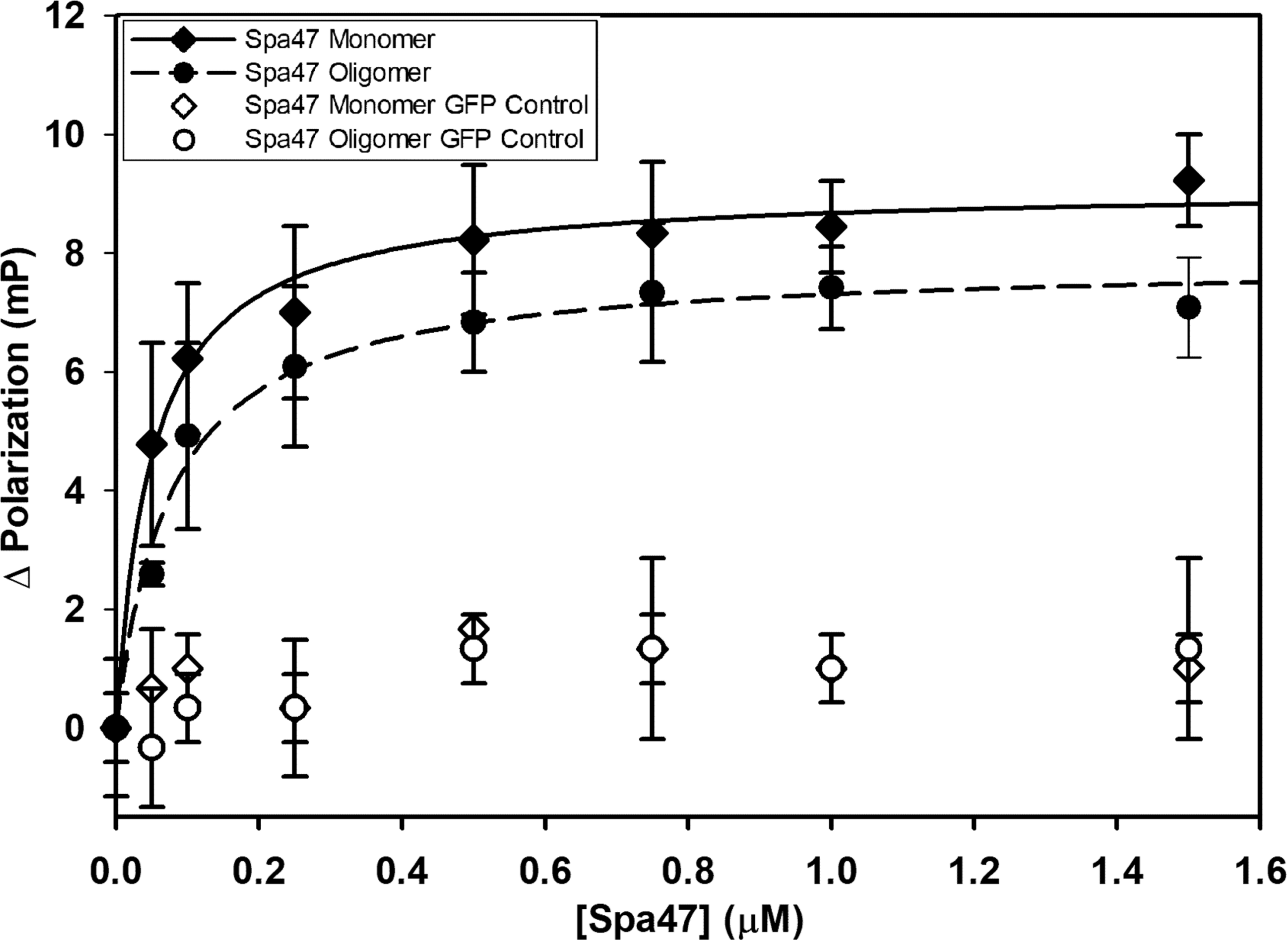
Spa33C interacts strongly with both monomeric and oligomeric Spa47. Fluorescence polarization assays were carried out between an engineered EGFP-Spa33C chimera and isolated monomeric and oligomeric Spa47 species. The polarization results were fit to a single-site saturation binding model using SigmaPlot12.0 with the data and fit for interaction between Spa33C and oligomeric Spa47 represented with filled diamonds and solid black fit line (R^2 = ^0.98) and the data for Spa33C interaction with monomeric Spa47 represented by filled circles and dashed fit line (R^2 = ^0.99). Control conditions in which monomeric or oligomeric Spa47 was titrated into purified EGFP did not result in a marked change in polarization (open shapes). Each graph was generated from triplicate technical replicates and is representative of a minimum of three independent experimental analyses used to determine the substrate concentration resulting in half maximum binding for each condition. Error bars represent the standard deviation around the mean.

The binding affinities of Spa33C to both monomeric and oligomeric Spa47 were calculated by fluorescence polarization. This first required engineering an EGFP-Spa33C fluorescent protein chimera as a probe for the assay. Importantly, the EGFP-Spa33C chimera not only expresses and purifies well from *E. coli*, but functions properly in vivo, as it complements *Shigella* virulence phenotype to 107 ± 14% of wild type when expressed in conjunction with Spa33FL in a Spa33 null strain (**Supplementary Figure S5**). Holding the concentration of the fluorescent Spa33C construct constant at 100 nM and titrating in either oligomeric or monomeric Spa47 (0 –1.5 μM) resulted in a sharp rise in polarization followed by saturation, as is expected for a strong binding event. The polarization data fit well to a single site saturation binding model with R^2^ values of 0.98 and 0.99 for Spa33C interaction with oligomeric Spa47 and monomeric Spa47, respectively (**Figure 3**). Binding affinity was measured by determining the concentration of Spa47 resulting in half-maximum binding, confirming high affinity interactions between Spa33C and both oligomeric and monomeric Spa47 (80 ± 10 nM and 50 ± 7 nM, respectively). These data provide the first quantitative description of interactions between Spa33C and Spa47 species and suggest that this high affinity interaction is in rapid equilibrium as the pull-down assay in **Supplementary Figure S2** resulted in modest levels of purified complex. EGFP was used as a control in place of Spa33C in which the purified EGFP concentration was held constant at 100 nM and both Spa47 species were titrated into the solution exactly as done for the experimental conditions. The results of the control assays are plotted in **Figure 3**, and it is clear that the EGFP is not driving an interaction with either Spa47 species.

### Spa33C differentially regulates Spa47 ATPase activity

**Figure 4:**
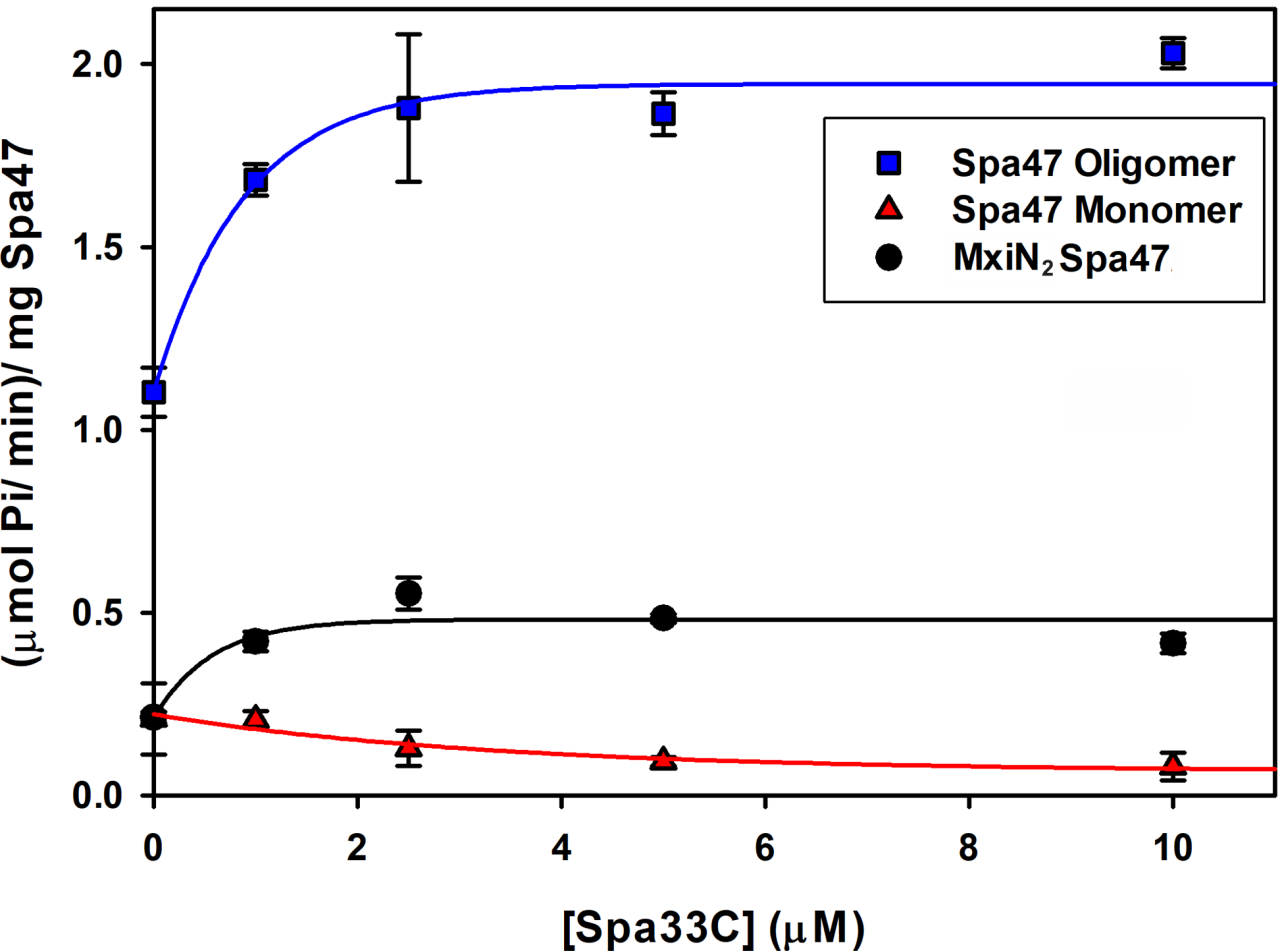
Spa33C differentially regulates the ATPase activity of monomeric and oligomeric Spa47. The effect of Spa33C on Spa47 activity was tested for oligomeric, monomeric, and co-expressed Spa47/MxiN. Oligomeric Spa47 activity increases with Spa33C concentration (blue squares), while monomeric Spa47 activity decreases with increasing concentrations of Spa33C (red triangles). The activity of the MxiN_2_Spa47 complex increases with the concentration of Spa33C (black circles). All data points are presented as the mean ± the standard deviation from three independent analyses.

**Figure 5:**
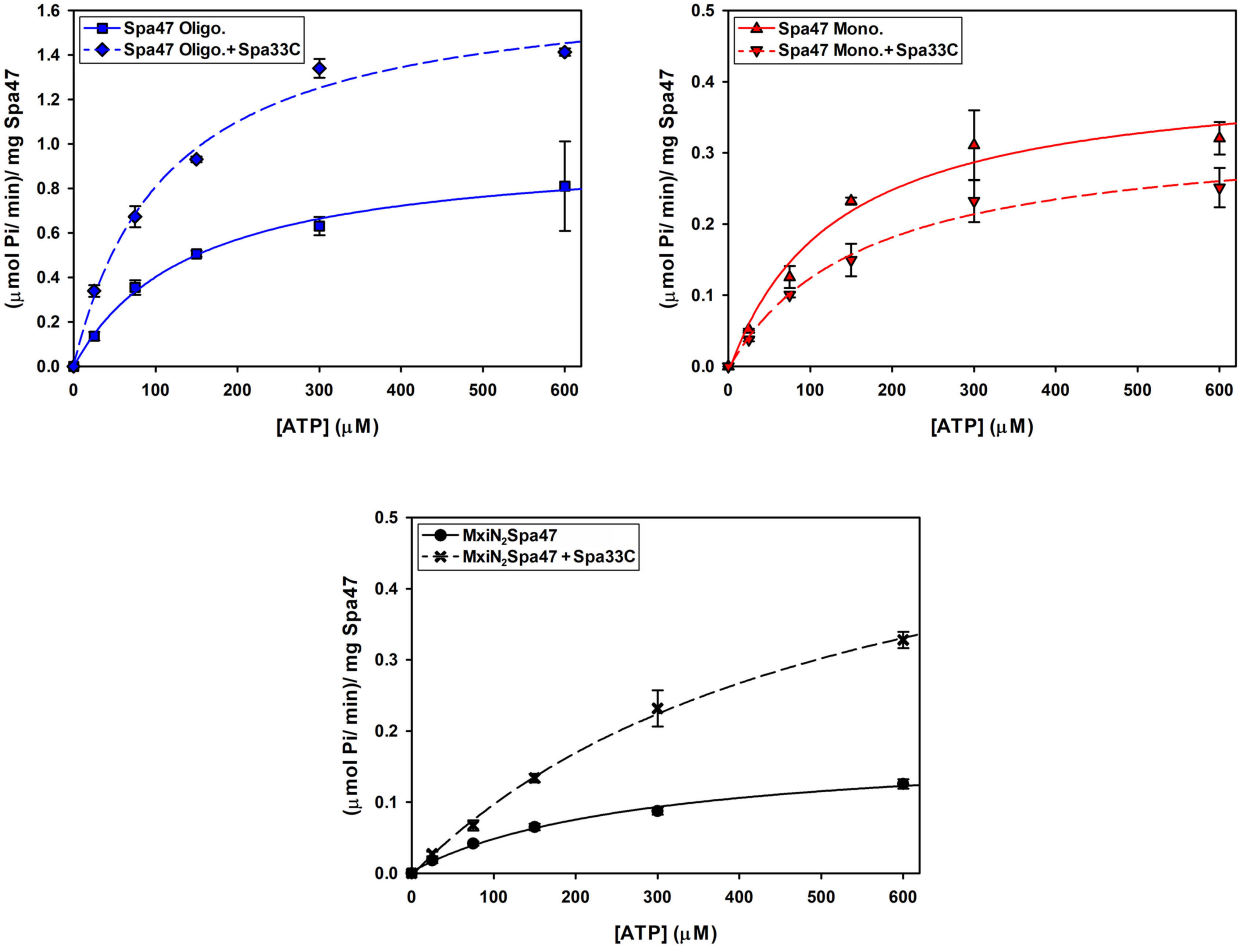
Kinetic analysis of the effect of Spa33C on Spa47 ATPase activity. The effect of Spa33C on monomeric Spa47, oligomeric Spa47, and MxiN_2_Spa47 substrate concentration dependence was tested by plotting initial reaction velocities as a function of substrate (ATP) concentration in the absence and presence of saturating concentrations of Spa33C and fitting the data to the Michaelis–Menten equation. All data points are presented as the mean ± standard deviation from three independent analyses.

Having confirmed that Spa33C interacts with Spa47, the effect of Spa33C on Spa47 ATPase activity was tested on both oligomeric and monomeric Spa47 species and on the MxiN_2_Spa47 heterotrimeric complex proposed to represent a chaperoned cytoplasmic species (Case and Dickenson, 2018). When oligomeric Spa47 ATPase activity was tested in the presence of increasing concentrations of Spa33C, the activity increased exponentially from 1.1 to 2.0 (µmol Pi/min)/mg Spa47, saturating with the addition of ~4 µM Spa33C (**Figure 4**). The addition of Spa33C to monomeric Spa47, however, had a more subtle and opposite effect, decreasing activity from 0.2 to 0.08 (µmol Pi/min)/mg Spa47. The addition of Spa33C to the purified MxiN_2_Spa47 complex increased the activity from 0.2 to 0.4 (µmol Pi/min)/mg Spa47 with a similar sensitivity to Spa33C concentration as observed for the homo-oligomeric Spa47 complex. As a control, the effect of bovine serum albumin (BSA) on the activity of the Spa47 oligomer, monomer, and the MxiN_2_Spa47 complex was also tested, confirming that the effect of Spa33C on Spa47 activity is specific and was not due to molecular crowding or non-specific protein–protein interactions (**Supplementary Figure S6**). To better understand the effect(s) of Spa33C on the activity of each Spa47 species, substrate-dependent kinetic analyses were performed on the Spa47 oligomer, monomer, and MxiN_2_Spa47 complex in the presence and absence of saturating concentrations of Spa33C (4 µM) (**Figure 5**). Initial velocity values for each condition were fit to the Michaelis–Menton equation, and the kinetic parameters K_M_, V_max_, k_cat_, and k_cat_/K_M_ were determined (**Table 1**). Interestingly, while the V_max_ and K_cat_ values tracked well with the impact on activity observed as a function of Spa33C concentration (**Figure 4**), the K_M_ was unaffected, suggesting that the regulatory mechanism impacts one or more rate- limiting steps that are independent of substrate concentration (perhaps formation of productive interfacial active sites). The addition of Spa33C to the MxiN_2_Spa47 complex increases the apparent V_max_ by a factor of 3.5 despite a marked increase in the apparent K_M_, the result of enhancing overall catalytic capability but reducing substrate sensitivity.

**Table 1:** Effect of oligomerization and Spa33C interaction on Spa47 enzyme kinetics.

Spa47 Construct^a^	K_M_^b^ (µM)	V_max_^c^ (µmol min^−1^ mg^−1^)	k_cat_^d^ (s^−1^)	k_cat_/K_M_^e^ (M^−1^ s^−1^)
Spa47 Oligomer	150 ± 20	0.98 ± 0.05	0.78 ± 0.04	(5.6 ± 0.9) × 10^3^
Spa47 Oligomer + Spa33C	120 ± 30	1.70 ± 0.1*	1.37 ± 0.09*	(12.0 ± 3.0) × 10^3^*
Spa47 Monomer	130 ± 50	0.42 ± 0.04	0.33 ± 0.03	(2.6 ± 0.9) × 10^3^
Spa47 Monomer + Spa33C	160 ± 40	0.34 ± 0.03*	0.27 ± 0.02*	(1.6 ± 0.5) × 10^3^
MxiN_2_Spa47	290 ± 70	0.18 ± 0.02	0.14 ± 0.02	(0.5 ± 0.1) × 10^3^
MxiN_2_Spa47 + Spa33C	530 ± 90*	0.63 ± 0.05*	0.50 ± 0.04*	(0.9 ± 0.2) × 10^3^*

a Initial reaction velocity was measured as a function of substrate (ATP) concentration for each of the Spa47 constructs described and the results fit to the Michaelis–Menten equation to determine the kinetic parameters for each condition.

b K_M_ is the Michaelis constant and represents the substrate concentration resulting in ½ maximum velocity.

c V_max_ is the maximum rate of the reaction.

d k_cat_ is V_max_ divided by the total enzyme concentration.

e k_cat_/K_M_ is the specificity constant and an overall measure of enzyme efficiency.

All values are presented as the mean ± standard deviation of three independent analyses.

*Indicates a significant difference from the equivalent Spa47 condition absent of Spa33C (Student’s one-tailed, unpaired t-test, p ≤ 0.05)).

### Spa33FL and Spa33C localize independently to the *Shigella* type three secretion system injectisome

**Figure 6:**
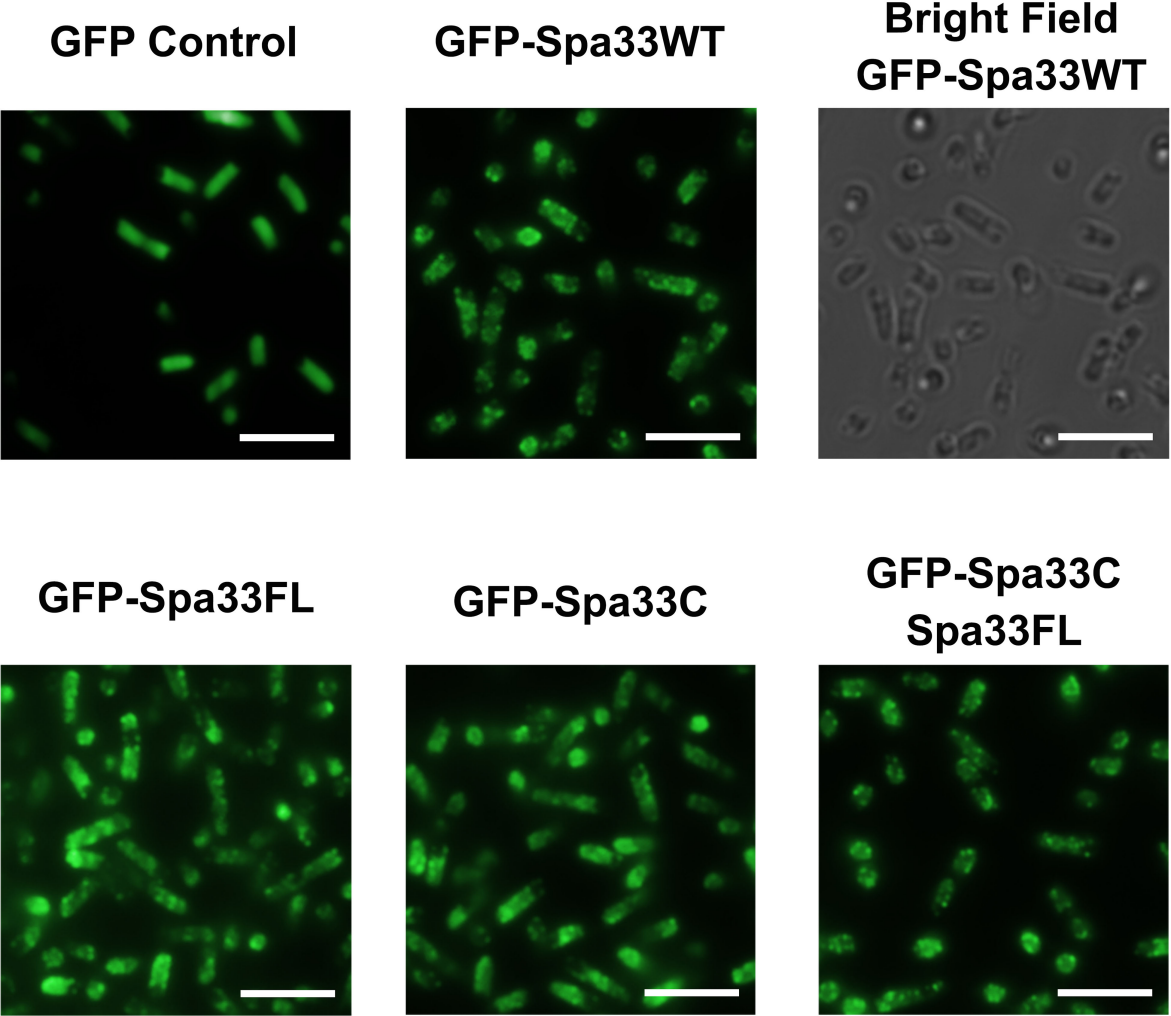
Total internal reflection fluorescence microscopy (TIRFM) localizes wild-type and variant Spa33 constructs to the *Shigella* T3SS. Expression of the EGFP-Spa33 variants used in this study resulted in punctate fluorescence distribution indicative of incorporation of the Spa33 chimeras into the injectisome sorting platform. Fluorescence from the EGFP control is diffuse throughout the cytoplasm. Scale bars = 5 μM.

To better understand the potential for the recently described Spa33C construct to incorporate into the T3SS injectosome, N-terminal EGFP-Spa33 chimeras of Spa33WT, Spa33FL only, Spa33C only, and a polycistronic construct encoding EGFP-Spa33C and Spa33FL were developed. Importantly, the addition of the N-terminal EGFP tag in these chimeras does not disrupt Spa33 function *in vivo* as *Shigella* strains expressing the fluorescent chimera complexes perform nearly identically to their native non-GFP counterparts in a cellular invasion assay (**Table 2** and **Figure 1**). The EGFP-Spa33 constructs were each expressed in *Shigella* to visualize their localization within the bacteria using live-cell total internal reflection fluorescence microscopy (TIRFM) as we had done previously to visualize punctate Spa47 distribution, as it incorporated into the sorting platform (Burgess et al., 2020). Unsurprisingly, expression of EGFP alone results in a homogeneous/diffuse distribution of EGFP throughout the cells, while the expression of EGFP-Spa33WT in a Spa33 null *Shigella* strain results in discrete punctate regions of fluorescence visible within the cells as the fluorescent Spa33FL chimera associates with T3SS injectisomes (**Figure 6**). It is important to note that in the EGFP-Spa33WT construct, only the Spa33FL protein contains a fluorescent N-terminal EGFP, as the naturally expressed Spa33C construct lacks the N-terminus, and while it is expressed, it lacks an EGFP tag. The distribution of N-terminal EGFP chimeras of Spa33FL and Spa33C was examined independently of one another and in conjunction with the unlabeled counterpart. Exploring these permutations found that both EGFP-Spa33FL and EGFP-Spa33C produce punctate localization (**Figure 6**), suggesting that they independently associate with the injectisome without requiring their Spa33 counterpart (FL or C), despite clearly requiring expression of both Spa33FL and Spa33C for proper T3SS function (**Figure 1**; **Supplementary Figure S1**, and **Table 2**).

**Table 2:** Effect of engineered N-terminal EGFP-Spa33 construct chimeras on *Shigella* invasion phenotype.

*Shigella* strain	Invasion^a^ (% ± SD)
Spa33WT	100
Spa33 null	0 ± 0*
EGFP-Spa33WT	116 ± 28
EGFP- Spa33FL only	15 ± 4*
EGFP-Spa33C only	1 ± 0*
EGFP-Spa33C/Spa33FL	107 ± 14

a The ability of the tested *Shigella* strains to invade cultured HeLa cells was measured by a standard gentamicin protection assay. Invasion results are presented as the percent ± standard deviation relative to the S. flexneri strain expressing Spa33WT (n = 3 independent measurements).

*Indicates a significant difference in invasion levels between the strain expressing wild-type Spa33 and the specified mutant (one-way ANOVA followed by a Dunnett’s post-test, p ≤ 0.05).

## Discussion

The work presented in this study uncovers a potentially novel T3SS regulatory pathway by characterizing the native C-terminal translation product of the T3SS protein Spa33 (Spa33C), finding that it is essential for proper T3SS function and *Shigella* virulence. As context, the full-length Spa33 protein and its SctQ family homologs in other T3SSs are essential C-ring components that universally support proper apparatus assembly (Notti and Stebbins, 2016). Interestingly, like Spa33, several homologs including HrcQ_B_ from *P. syringae*, YscQ from *Y. pseudotuberculosis*, and SpaO from *S. enterica* each result in the production of in-frame C-terminal translation products of the parent genes (Fadouloglou et al., 2004; Bzymek et al., 2012; Lara-Tejero et al., 2019). By and large, the specific role(s) of these truncated products have remained enigmatic, and this has been compounded by starkly different phenotype outcomes resulting from mutant strains in which they have been eliminated. More specifically, the expression of both full- length and truncated SctQ translation products is essential for T3SS effector protein secretion in *Y. pseudotuberculosis* (Bzymek et al., 2012) and *Shigella* (McDowell et al., 2016; Kadari et al., 2019) (**Supplementary Figure S1**) and for *Shigella* invasion phenotype (**Figure 1**). Preventing expression of the shortened C-terminal SpaO construct in *Salmonella*, however, only slightly reduces the efficiency of apparatus formation and effector protein secretion, leading the authors to suggest that the abbreviated SpaO construct is not a core component of the sorting platform in *Salmonella*, but may play a role in regulating platform assembly during type three secretion (Lara-Tejero et al., 2019). These contradictory findings are perhaps at the heart of the divergent effects of the truncated SctQ proteins on T3SS function in different bacteria, as it seems quite clear that Spa33C does interact directly with the *Shigella* T3SS, even suggested recently to complex with Spa33FL, fulfilling homologous structural roles of the flagellar C-ring FliM/FliN-FliN components, respectively (McDowell et al., 2016). The functional similarities between them remain obscured, however, as FliM and FliN play essential roles not only in flagellar assembly but also in torque generation and direction switching of the flagellum, and no such evidence for rotation exists in the non-flagellar virulence T3SS apparatus, ruling out similar function for Spa33C. Regardless, the co-expression/co-purification of Spa33C with the sorting platform proteins Spa33FL, MxiN, and Spa47 (**Figure 2**) and the observed association of both Spa33FL and Spa33C GFP chimeras with the sorting platform (**Figure 6**) are indicative of strong association of both constructs with (if not incorporation into) the *Shigella* T3SSs sorting platform where they act cooperatively in support of proper T3SS function (**Figure 1**; **Table 2**; **Supplementary Figure S1**).

In addition to this clear reliance of *Shigella* on Spa33C for T3SS function, perhaps the most exciting finding from this work is uncovering the regulatory role of Spa33C with respect to Spa47 ATPase activity. Like the previously described regulatory protein MxiN (Case and Dickenson, 2018), Spa33C interacts strongly with both monomeric and oligomeric populations of Spa47 (**Figure 3**) and differentially affects ATPase activity of the two Spa47 populations (**Figures 4**, **5**; **Table 1**) with opposite impact on activity from MxiN. In contrast to MxiN, which forms a highly stable MxiN_2_Spa47 heterotrimer, Spa33C interaction with Spa47 appears to be transient, resulting in sub-100 nM affinities in a fluorescence polarization assay (**Figure 3**), but only modest complex co-purification in a pull-down assay (**Supplementary Figure S2**). This type of transient interaction supported by a rapid equilibrium would be ideally suited for generating a rapid response by Spa47 within the sorting platform to changing cytoplasmic Spa33C conditions. Moreover, Spa33C also upregulates the activity of the chaperoned MxiN_2_Spa47 complex, which already displays enhanced activity compared to the isolated Spa47 monomer (Case and Dickenson, 2018), perhaps playing a cooperative role with MxiN in preparing the complex for incorporation into the apparatus and activation of protein secretion and/or in directly regulating the activity of MxiN_2_Spa47, as it has been suggested to shuttle substrates to the injectisome where ATP hydrolysis drives chaperone release and facilitates secretion (Akeda and Galan, 2005).

Together, this work demonstrates the importance of Spa33C in *Shigella* T3SS function and virulence and describes only the second differential regulator of T3SS ATPase activity to date. It cannot be overstated that having identified both regulators in *Shigella* provides a unique opportunity to explore the idea that MxiN and Spa33C not only play critical structural roles in the injectisome sorting platform, but that they also provide a necessary yin and yang that controls virulence through T3SS activity and Spa47-catalyzed ATP hydrolysis in response to currently unidentified stimuli and response pathways. Uncovering the specifics of these pathways will provide answers to the long-standing question of how T3SS activity is regulated *in vivo* and will identify much needed targets for non-antibiotic therapeutics geared toward the treatment of the growing number of antibiotic-resistant strains of *Shigella* and other T3SS expressing pathogens that likely utilize similar regulatory pathways.

## Data availability statement

The raw data supporting the conclusions of this article will be made available by the authors, without undue reservation.

## Author contributions

HC and ND contributed to the conception and design of the study. HC, SG, MG, and ND all contributed to data collection and interpretation. HC wrote the first draft of the manuscript. HC and ND wrote sections of the manuscript. All authors contributed to manuscript revision, read, and approved the submitted version.
