# Supplementary material for: Differential regulation of Shigella Spa47 ATPase activity by a native C-terminal product of Spa33
Source: Front Cell Infect Microbiol. 2023 Jun 14;13:1183211. doi: 10.3389/fcimb.2023.1183211 (PMC10302723; doi:10.3389/fcimb.2023.1183211)
Supplement: Supplementary file 1 [file DataSheet_1.pdf]

Supplementary Material

Differential Regulation of *Shigella* Spa47 ATPase Activity by a Native C-terminal Product of Spa33

Heather B. Case, Saul Gonzalez, Marie E. Gustafson, and Nicholas E. Dickenson*

*** Correspondence:** Corresponding Author: nick.dickenson@usu.edu

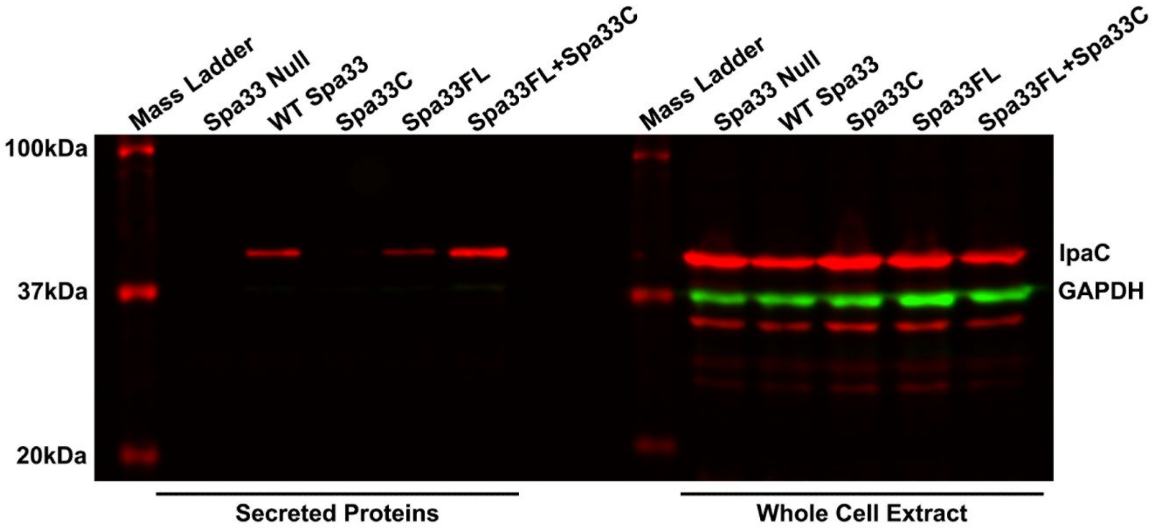

Supplementary Figure S1. Immunoblot detection of the translocator protein IpaC in Congo Red-induced *Shigella* Spa33 mutants. Robust levels of IpaC are observed in the whole cell extracts of each strain while IpaC secretion is highly dependent upon the Spa33 constructs expressed in the engineered mutants. The cytoplasmic enzyme GAPDH serves as a loading and cytoplasmic control.

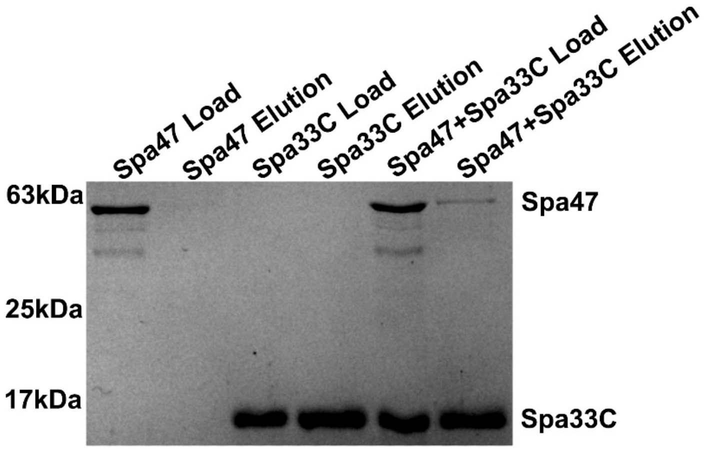

Supplementary Figure S2. Pull-down assay between Spa33C and Spa47. Spa33C containing an N-terminal 6 x Histidine tag and Spa47 containing no affinity tag were exposed to Ni-NTA resin independently and after incubating together for 30 minutes. The load solution and elution fraction for each condition were run on an SDS-PAGE gel and the proteins visualized using Coomassie brilliant blue. The presence of Spa47 in the elution fraction resulting from the Spa47/Spa33C solution but not in the elution fraction from the Spa47 only condition suggests it is purified as part of a Spa47/Spa33C complex.

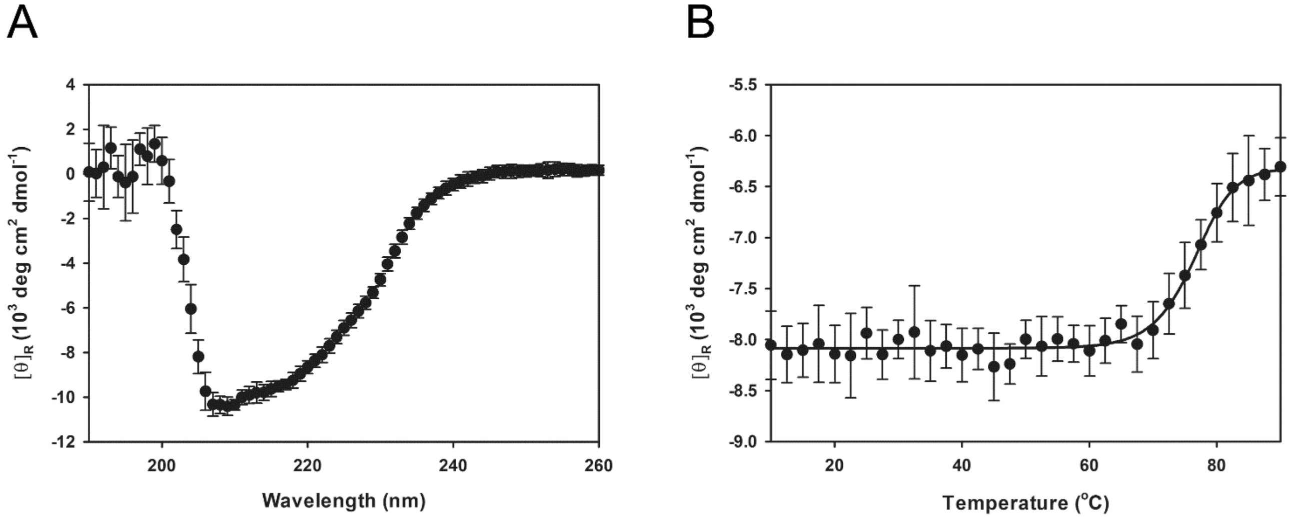

Supplementary Figure S3. Analysis of the secondary structure content and stability of Spa33C. (A) Far-UV CD spectra of Spa33C. **(B)** Thermal unfolding of the secondary structure of Spa33C is observed by plotting the mean residue molar ellipticity at 222 nm while the protein solution was heated from 10 to 90 °C. The data are plotted as the mean ± the standard deviation from three independent measurements.

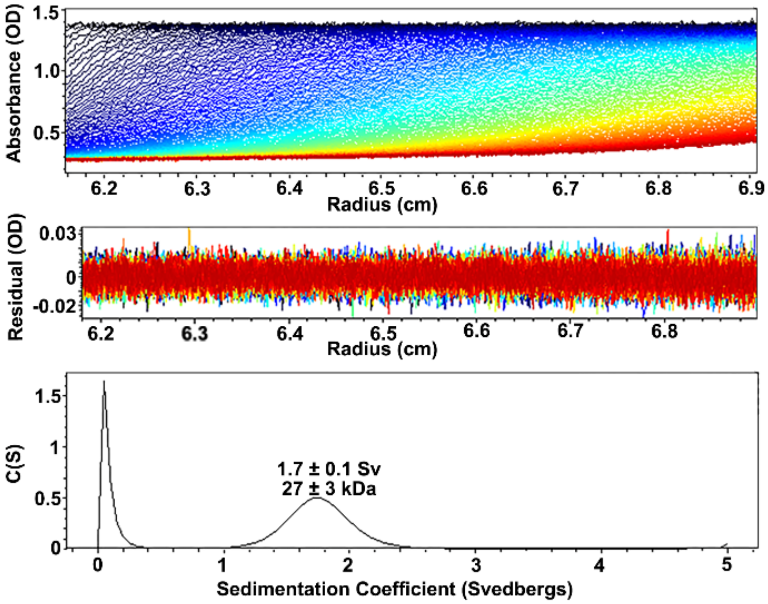

Supplementary Figure S4. AUC analysis of Spa33C. A_{280} absorbance scans of Spa33C were monitored during SV-AUC. Color gradation visualizes the time course of collected scans. Representative residuals from fitting the data to a continuous c(s) distribution model are shown as well as sedimentation coefficient distributions (c(s) versus S). Spa33C sediments primarily as a single species with a sedimentation coefficient of 1.7 ± 0.1 Svedbergs, consistent with a Spa33C homo-dimer. The displayed sedimentation coefficient and calculated molecular weight are presented as the mean ± standard deviation of three independent measurements of Spa33C sedimentation performed at three different concentrations to ensure the sedimentation results are consistent and concentration-independent.

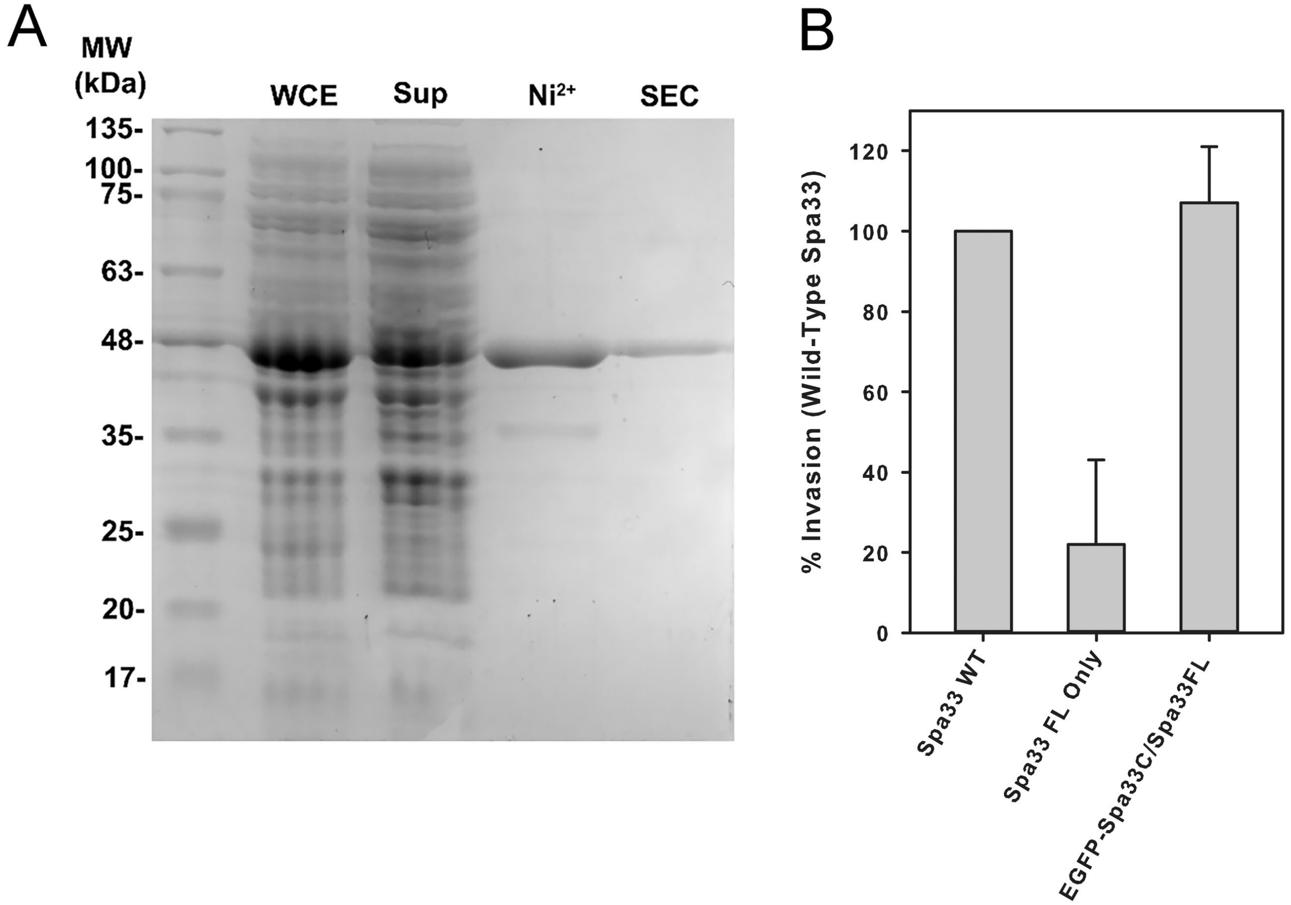

Supplementary Figure S5. Characterization of the EGFP-Spa33C chimera developed for this study. A) An SDS-PAGE gel of the whole cell extract (WCE) of *E. coli* expressing the engineered EGFP-Spa33C chimera, the supernatant resulting from isolation of soluble proteins in the WCE (Sup), a representative elution fraction following Ni^{2+} affinity purification, and a representative elution fraction following size exclusion chromatography. **B)** A cellular invasion assay confirms expression of the EGFP-Spa33C chimera complements *Shigella* invasion phenotype. The full invasion dataset is available in Table 2 in the main text.

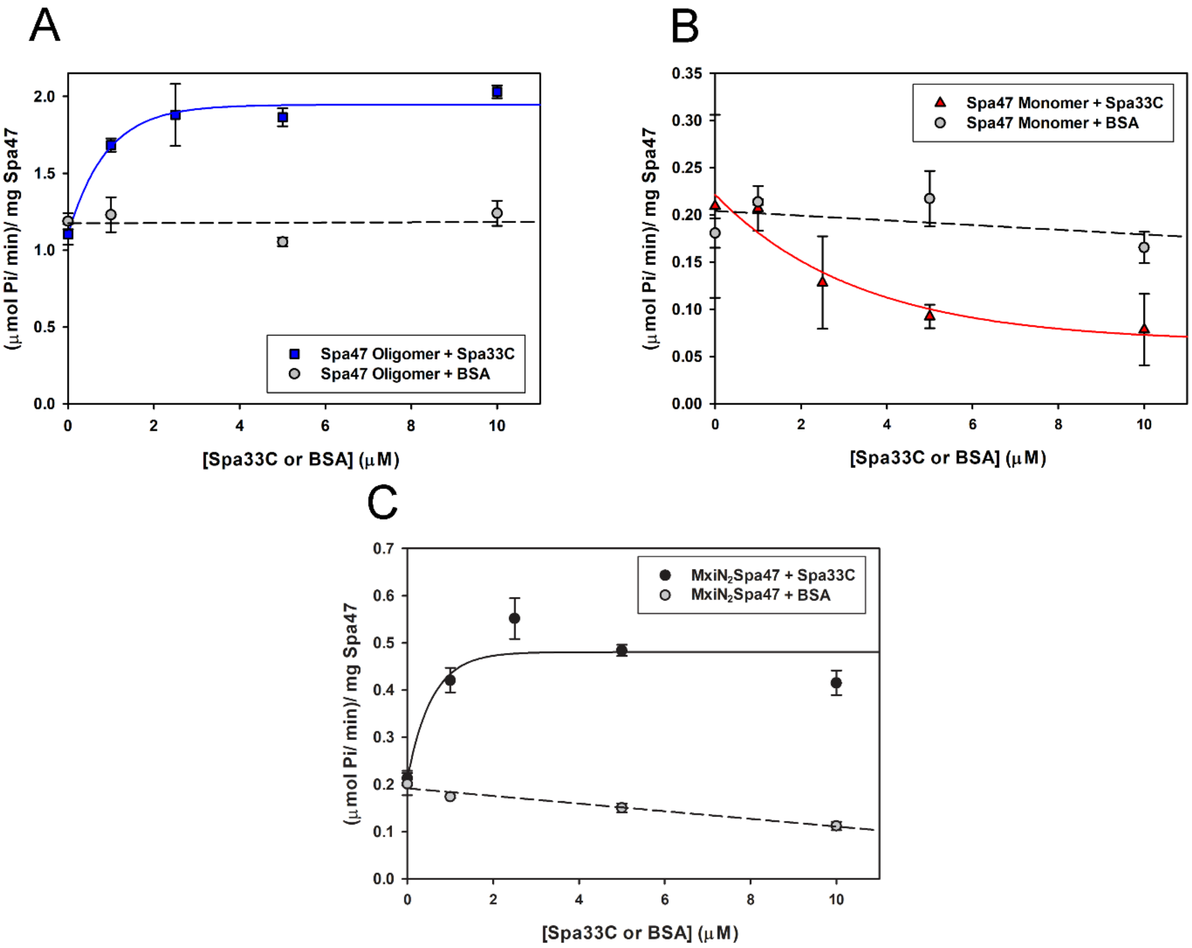

Supplementary Figure S6. Spa47 activity profiles as a function of Spa33C and BSA concentration. The effect of Spa33C on the activity of **A)** Spa47 oligomer, **B)** Spa47 monomer, and **C)** co-expressed MxiN2Spa47 was tested. Bovine serum albumin was additionally substituted for Spa33C and plotted as a control to ensure that the observed effects of Spa33C on Spa47 are not due to molecular crowding or non-specific protein interactions. The addition of BSA did not significantly affect the rate of ATP hydrolysis of any of the tested Spa47 constructs, ensuring that the effects seen by Spa33C are specific.

Supplementary Table S1. Circular Dichroism analysis of Spa33C[a]

α-Helix	13 ± 1 %
β-Sheet	33 ± 2 %
Turns	25 ± 1 %
Unstructured	29 ± 1 %
Tm[b]	77 ± 1 °C

[a]Secondary structure content analysis of far-UV CD spectra was performed using the Dichroweb software package CDSSTR. The determined percent α-helix, β-sheet, turns, and unstructured regions are reported for Spa33C. [b]Secondary structure thermal stability was tested by measuring CD signal at 222 nm while increasing the solution temperature from 10 to 90 °C. Tm values were determined by identifying the inflection point of each major transition to fully unfolded protein. Predicted secondary structure content and Tm values are reported as the mean ± standard deviation of the analyses from three independent spectra/profiles.

Supplementary Table S2. DNA Primers Used In This Study

Gene	*Forward/Reverse*	*Purpose*	*Sequence*
MxiN	Forward	Add 5' NdeI	5'-gagagagagCATATGcaaaaaggcactttacc
MxiN	Reverse	Add 3' BamHI	5'-gagagagagGGATCCttaatcattaacaggattctctt
MxiN	Forward	Add 5' BamHI	5'-gagagagagGGATCCatgcaaaaaggcactttac
MxiN	Reverse	Add 3' HindIII	5'-gagagagagAAGCTTttaatcattaacaggattctctttt
Spa33	Forward	Add 5' NdeI	5'-agagagagaCATATGctaagaattaaacattttg
Spa33	Reverse	Add 3' XhoI	5'-gagagagagCTCGAGttactcctttaccatccaa
Spa33	Forward	Mutate internal RBS	5'-tgattatattgtgaatgataataatgagg
Spa33	Reverse		5'ccaataataacttgattacacaataataaa
Spa33	Forward	Mutate alternate start codon	5'-caatgataataatgaggcaaaaattaatc
Spa33	Reverse		5'-acaatataatcaccaataataacttgat
Spa33	Forward	Truncate Spa33 to Spa33C<br>Add 5' NdeI	5'-agagagagaTCTAGAatgaatgataataatgaggc
Spa33	Forward	Truncate Spa33 to Spa33C<br>Add 5' BamHI	5'-agagagagaGGATCCatgaatgataataatgaggc
Spa33	Forward	Add 5' XbaI	5'-agagagagaTCTAGAatgctaagaattaaacattttgacgc
Spa33	Forward	Add 5' XbaI to Spa33C	5'-agagagagaTCTAGAatgaatgataataatgaggcaaaaattaatctg
Spa33	Reverse	Add 3'PstI	5'-ggtggttCTGCAGttactcctttaccatccaagaac

Supplementary Methods

Evaluation of Congo Red-induced T3SS activation. The engineered Spa33 *Shigella* mutants described in this study were grown at 37 °C in tryptic soy broth containing appropriate antibiotics to an OD_{600} of 1.0 prior to isolation by centrifuging the cultures and rinsing the resulting cell pellets to remove extracellular proteins. The bacteria were then incubated in sodium phosphate buffer containing 0.28 mg/mL Congo Red at 37 °C for 30 min in to activate the T3SS and initiate effector secretion. The cultures were centrifuged at 13,000× g for 15 min to isolate the supernatant containing secreted effector proteins. The proteins in the isolated cell pellets and supernatants were separated via SDS-PAGE, transferred to PVDF membranes by Western blot, and probed with α-IpaC rabbit polyclonal antibodies and Alexa 647 goat α-rabbit secondary antibodies. The rabbit polyclonal α-IpaC antibodies have been previously validated for specific detection of IpaC in a similar Congo Red secretion assay.[1] Antibodies against glyceraldehyde 3-phosphate dehydrogenase (GAPDH) provided loading and lysis controls.

Far-Ultraviolet (Far-UV) Circular Dichroism (CD)- Far-UV CD spectra and thermal stability profiles were obtained for Spa33C. Measurements were taken using a JASCO model J-1500 spectropolarimeter equipped with a six-position sample holder and a Peltier temperature controller (Jasco, Easton, MD). Spectra were collected from 190 nm to 260 nm at 10 °C using 0.1 cm quartz cuvettes, 1 nm data sampling, a 50 nm/min scan rate, and a 2 sec data integration time. Secondary structure thermal stability profiles were collected in the same 0.1 cm quartz cuvettes by monitoring the CD signal at 222 nm while the solution temperature was increased from 10 °C to 90 °C at a rate of 0.3 °C/min. CD analysis was performed on 0.3 mg/mL protein for Spa33C and was evaluated in 20 mM Tris, 100 mM NaCl, 5% (v/v) glycerol, and 5 mM DTT (pH 7.9). CD signals were converted to mean residue molar ellipticity and secondary structure content analysis was performed using the Dichroweb software[2,3] and CDSSTR analysis package.[4] Thermal unfolding transition temperatures (Tm) were determined by plotting the derivative of each thermal unfolding curve and identifying the corresponding local maxima.

Analytical ultracentrifugation- Sedimentation velocity analytical ultracentrifugation (SV-AUC) experiments were conducted using an Optima XL-I (Beckman Coulter, Fullerton, CA) analytical ultracentrifuge equipped with scanning UV/visible optics. A 50 Ti eight-hole rotor and cells with Beckman 12 mm path length charcoal-epon two sector centerpieces and quartz windows were used. Protein samples were analyzed at 20 ˚C and 40,000 RPM using absorbance detection at 280 nm and scanning until complete sedimentation was achieved. Scans were performed at multiple protein concentrations to ensure that the obtained results were concentration-independent (6.5 µM 14 µM, and 21 µM). The data were analyzed using a continuous c(s) distribution and SEDFIT version 15.01b.[5] The buffer density (1.0174 g/mL), buffer viscosity (0.01822 poise), and the Spa33C partial specific volume (0.732143 mL/g) were calculated using Sednterp version 20130813 BETA.[6]

Spa33C/Spa47 pull-down assay- An *in vitro* pull-down assay was performed between purified Spa47 containing no purification tag and Spa33C expressed with an N-terminal 6 X Histidine tag. Solutions containing 2.5 µM Spa47, 8 µM Spa33C, and 2.5 µM Spa47 combined with 8 µM Spa33C were prepared in binding buffer (20 mM Tris, 100mM NaCl, 5% glycerol, pH 7.9) and incubated for 30 minutes at room temperature. Each protein solution was exposed to 100 µL of Ni^{2+} charged IMAC resin, copiously rinsed with binding buffer, and bound protein eluted with elution buffer (20 mM Tris, 100 mM NaCl, 400 mM imidazole, 5% glycerol, pH 7.9). Proteins present in the load conditions and elution fractions visualized via SDS-PAGE to qualitatively assess interaction.

1. Burgess JL, Burgess RA, Morales Y, Bouvang JM, Johnson SJ, Dickenson NE. Structural and Biochemical Characterization of Spa47 Provides Mechanistic Insight into Type III Secretion System ATPase Activation and Shigella Virulence Regulation. J Biol Chem. 2016;291(50):25837-52. PMC5207059.
2. Lobley A, Whitmore L, Wallace BA. DICHROWEB: an interactive website for the analysis of protein secondary structure from circular dichroism spectra. Bioinformatics. 2002;18(1):211-2.
3. Whitmore L, Wallace BA. DICHROWEB, an online server for protein secondary structure analyses from circular dichroism spectroscopic data. Nucleic Acids Res. 2004;32(Web Server issue):W668-73. PMC441509.
4. Johnson WC. Analyzing protein circular dichroism spectra for accurate secondary structures. Proteins. 1999;35(3):307-12.
5. Lebowitz J, Lewis MS, Schuck P. Modern analytical ultracentrifugation in protein science: a tutorial review. Protein Sci. 2002;11(9):2067-79. PMC2373601.
6. Laue TM, Shah, B.D., Ridgeway, T.M. and Pelletier, S.L. Analyticall Ultracentrifugation in Biochemistry and Polymer Science. In: Rowe SHaA, editor.: Royal Society of Chemistry; 1992. p. 90-125.
